# Human Recombinant Peptide Sponge Enables Novel, Less Invasive Cell Therapy for Ischemic Stroke

**DOI:** 10.1155/2018/4829534

**Published:** 2018-04-08

**Authors:** Michiyuki Miyamoto, Kentaro Nakamura, Hideo Shichinohe, Tomohiro Yamauchi, Masaki Ito, Hisayasu Saito, Masahito Kawabori, Toshiya Osanai, Tasuku Sasaki, Kiyohiro Houkin, Satoshi Kuroda

**Affiliations:** ^1^Department of Neurosurgery, Hokkaido University Graduate School of Medicine, Sapporo, Japan; ^2^Department of Neurosurgery, Teikyo University Hospital, Tokyo, Japan; ^3^Regenerative Medicine Research Laboratories, Fujifilm Corporation, Kanagawa, Japan; ^4^Division of Clinical Research Administration, Hokkaido University Hospital, Sapporo, Japan; ^5^Department of Neurosurgery, Graduate School of Medicine and Pharmacological Science, University of Toyama, Toyama, Japan

## Abstract

Bone marrow stromal cell (BMSC) transplantation has the therapeutic potential for ischemic stroke. However, it is unclear which delivery routes would yield both safety and maximal therapeutic benefits. We assessed whether a novel recombinant peptide (RCP) sponge, that resembles human collagen, could act as a less invasive and beneficial scaffold in cell therapy for ischemic stroke. BMSCs from green fluorescent protein-transgenic rats were cultured and Sprague–Dawley rats were subjected to permanent middle cerebral artery occlusion (MCAo). A BMSC-RCP sponge construct was transplanted onto the ipsilateral intact neocortex 7 days after MCAo. A BMSC suspension or vehicle was transplanted into the ipsilateral striatum. Rat motor function was serially evaluated and histological analysis was performed 5 weeks after transplantation. The results showed that BMSCs could proliferate well in the RCP sponge and the BMSC-RCP sponge significantly promoted functional recovery, compared with the vehicle group. Histological analysis revealed that the RCP sponge provoked few inflammatory reactions in the host brain. Moreover, some BMSCs migrated to the peri-infarct area and differentiated into neurons in the BMSC-RCP sponge group. These findings suggest that the RCP sponge may be a promising candidate for animal protein-free scaffolds in cell therapy for ischemic stroke in humans.

## 1. Introduction

Stroke is a leading cause of death and disability but few treatment options exist despite intensive research [1]. Recent studies have strongly suggested that cell therapy can promote functional recovery of patients with various central nervous system (CNS) diseases, including ischemic stroke. Bone marrow stromal cells (BMSCs) are considered as candidates for donor cells because of their regenerative potential. BMSCs can survive in the infarcted brain, migrate towards the ischemic lesion, express neural phenotypes, and promote functional recovery when transplanted into animal stroke models [2, 3].

However, several problems remain to be solved before launching the clinical application of BMSCs for stroke. Issues include the optimal route of donor cell delivery in the clinical situation [2]. It is essential to determine the most desirable, least invasive route of cell delivery with maximal therapeutic effects prior to clinical application of cell therapy [4]. For example, BMSCs can be transplanted into injured CNS tissue through intravenous, intra-arterial, intracerebral, or intrathecal routes. Although intracerebral injection permits the most efficient delivery of donor cells to the damaged tissue, a less invasive procedure would be optimal [2]. Intravenous or intrathecal transplantation are attractive because they are less invasive, safe techniques for the host CNS, but they result in less pronounced cell migration and functional recovery than direct cell transplantation [5]. Alternatively, the intra-arterial injection of BMSCs may be feasible to less invasively deliver them to the damaged CNS [6]. However, there are a limited number of studies that directly compare the therapeutic effects of these delivery routes under the same conditions [2].

Recently, tissue engineering has developed into a promising cell delivery method. Tissue engineering aims to provide three-dimensional (3D) constructs to serve as replacement tissues or organs by combining donor cells and scaffolds. We have already reported the application of some biomaterial scaffolds in cell therapy for animal CNS disease models. First, we reported that fibrin matrix, which is widely used as a surgical glue, provides a suitable scaffold for BMSCs transplanted to rat spinal cord injury model and rat MCA occlusion model [7, 8]. Next, we reported use of a thermoreversible gelation polymer hydrogel for BMSC transplantation to a mouse MCA occlusion model [9]. We also reported the application of cell sheet technology to BMSC transplantation for rat brain infarcts, although, strictly speaking, a cell sheet is different from a scaffold [10]. Each of these tissue-engineering methods had its merits and issues.

In the present study, we assessed whether a novel recombinant peptide (RCP) sponge made of human collagen could act as a less invasive and beneficial scaffold in cell therapy for ischemic stroke.

## 2. Materials and Methods

### 2.1. Preparation of Rat BMSCs and RCP Sponges

**Figure 1:**
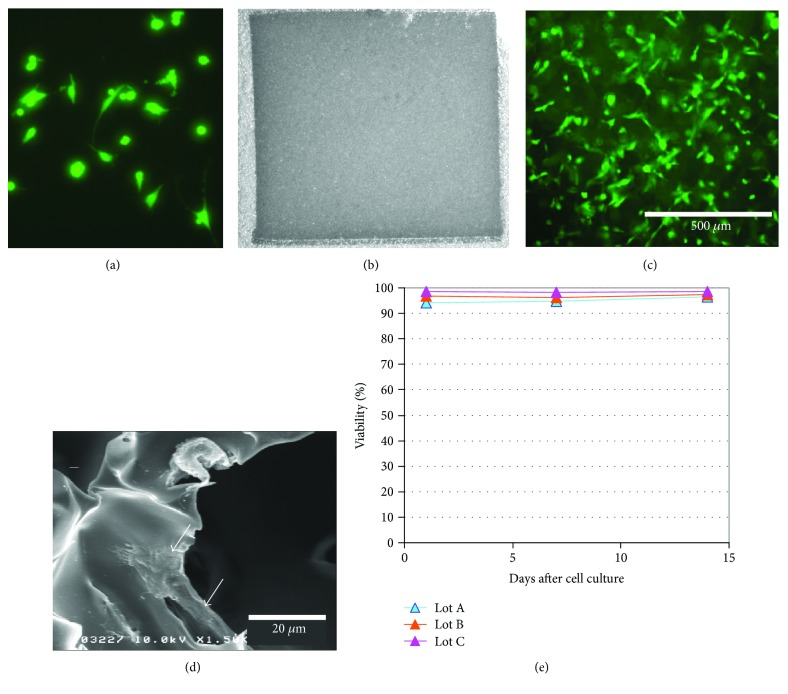
Analysis of RCP sponges with cultured BMSCs. Panel (a) shows a fluorescence photomicrograph of cultured rat GFP-BMSCs. Panels (b) and (c) show photomicrographs of the RCP sponge before cell culture (b) and 14 days after the second cell passage (c). Panel (d) shows a photomicrograph of BMSCs on the RCP sponge, using scanning electron microscopy. Panel (e) shows the line graph of the cell viability in RCP sponge. The arrow in (d) indicates a surviving BMSC on the RCP sponge. Scale bars: 500 *μ*m (c) and 20 *μ*m (d).

All animal experiments were approved by the Animal Studies Ethical Committee of Hokkaido University Graduate School of Medicine. The BMSCs were isolated under sterile conditions from the femurs of male 7-week-old transgenic Sprague–Dawley (SD) rats expressing enhanced green fluorescent protein (GFP; Japan SLC Inc., Hamamatsu, Japan), as described previously [11]. Whole marrow cells were placed in tissue culture flasks coated with collagen I (Becton Dickinson Labware, UK) with a medium that consisted of Dulbecco's modified Eagle's medium (DMEM; D6429, Sigma-Aldrich, Japan) containing 10% fetal bovine serum (FBS), 2 mM L-glutamine, and 100 U/mL penicillin. After 24 h, the nonadherent cells were removed by changing the medium. The culture medium was replaced 3 times per week (Figure 1(a)).

The RCP sponge, which was made from human collagen recombinant peptide, was supplied by Fujifilm Corporation (Kanagawa, Japan; Figure 1(b)). The RCP sponge was obtained using a previously reported method [12]. Freeze-dried RCP sponge was prepared using a 4% aqueous RCP solution that was gradually cooled to −40°C in a polytetrafluoroethylene chamber (TaKaRa Freeze-Dryer TF5-85ATNNN; TaKaRa Co. Ltd., Tokyo, Japan). Then the sponge was thermally cross-linked under reduced pressure at 160°C for 24 h. BMSCs (1 × 10^5^ cells in 50 mL of medium), which had been passaged twice, were seeded onto RCP sponges and incubated for 1 h, and then the sponges with BMSCs were cultured for 14 days under the conditions described above. The cells were detached using 0.05% trypsin-ethylenediaminetetraacetic acid (EDTA; Gibco, Hayward, CA, USA) and counted to evaluate the viability of BMSCs on the RCP sponge. The cell viability was checked on day 1, 7, and 14 postculture in RCP sponge.

### 2.2. Scanning Electron Microscope Analysis of BMSCs in RCP Sponge

A scanning electron microscope (HITACHI S-4500, Tokyo, Japan) was employed to observe the morphology of BMSCs in the RCP sponge. RCP sponges with BMSCs were fixed with 2.5% glutaraldehyde, dehydrated using ethanol, and then dried using isoamyl acetate. Finally, they were coated by ion sputtering and observed using electron microscopy.

### 2.3. Permanent Middle Cerebral Artery Occlusion Model

Male 7-week-old SD rats were purchased from CLEA Japan Inc. (Tokyo, Japan). Permanent middle cerebral artery (MCA) occlusion was performed as described previously [11]. Anesthesia was induced with 4.0% isoflurane in N_2_O : O_2_ (70 : 30) and maintained with 2.0% isoflurane in N_2_O : O_2_ (70 : 30). Core temperature was maintained at between 36.5 and 37.0°C throughout the procedure. Both common carotid arteries were exposed through a ventral midline incision of the neck, and then a 1.5 cm vertical skin incision was made between the right eye and ear. The temporal muscle was scraped from the temporal bone and a 5 × 5 mm temporal craniotomy was performed using a small dental drill. To prevent cerebrospinal fluid leakage, the dura mater was carefully kept intact, and the right MCA was ligated using a 10-0 nylon thread through the dura mater. The cranial window was then closed with the temporal bone flap and the temporal muscle and skin were sutured with a 4-0 nylon thread. Subsequently, the bilateral common carotid arteries were occluded by surgical microclips for 1 h. Animals that circled toward the paretic side after surgery were used for transplantation [13].

### 2.4. BMSC Transplantation

**Figure 2:**
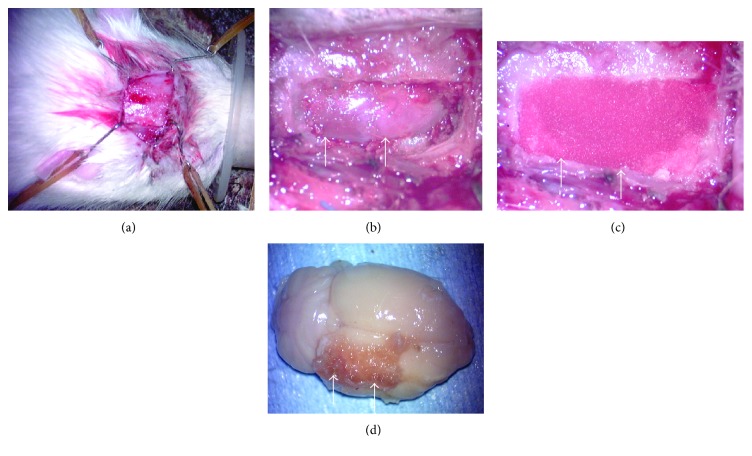
Cell transplantation in the BMSC/scaffold group. Panels (a), (b), and (c) show photographs of the surgical treatment for cell transplantation in the BMSC/scaffold group. Panel (d) shows a brain removed 35 days posttransplantation in the BMSC/scaffold group. Arrows indicate the outer rim of the craniotomy (b), the RCP sponge with BMSCs (c), and remaining sponge (d).

The animals subjected to permanent MCA occlusion were randomly divided into three groups: the BMSC/scaffold group, the BMSC/direct injection group, and the vehicle/direct injection group. Surgery for cell transplantation was performed 7 days postischemia in all groups. In the BMSC/scaffold group (*n* = 10), the cranium was exposed through a coronal skin incision (Figure 2(a)), and the brain surface was carefully exposed through a craniotomy (10 × 20 mm) made with a dental drill under a surgical microscope at the right parietal region adjacent to the infarcted brain (Figure 2(b)). The dura matter was carefully folded and the sheet of RCP sponge with GFP-expressing BMSCs was gently placed onto the brain surface (Figure 2(c)). Then, the cranial window was closed with the dura matter and the parietal bone flap and the surgical wound was closed.

**Figure 3:**
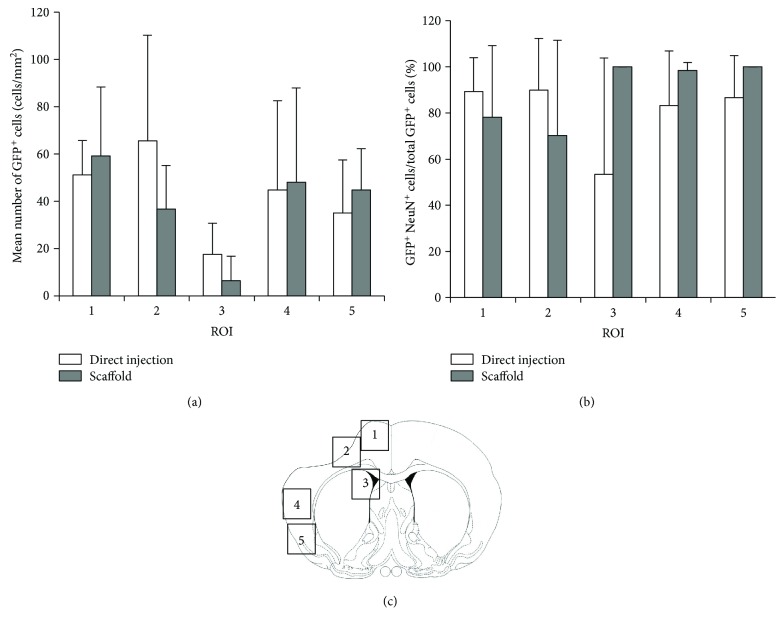
Histological analysis. The bar graphs show the mean number of GFP-positive cells (a) and the percentage of GFP and NeuN double-positive cells in the total GFP-positive cell population (b) in each ROI. Panel (c) indicates the location of each ROI. White bars: the BMSC/direct injection group; shaded bars: the BMSC/scaffold group. Error bars show SD.

GFP-BMSCs or vehicle were stereotactically transplanted into the ipsilateral striatum in the BMSC/direct injection group or the vehicle/direct injection group (*n* = 11 in each group), as reported previously [11]. Briefly, a burr hole was made 3 mm to the right of the bregma. A Hamilton syringe was inserted 5 mm from the surface of the dura mater into the brain parenchyma, and 10 *μ*L of cell suspension (1 × 10^6^ cells) or vehicle was introduced into the striatum (Figure 3(c)).

### 2.5. Behavioral Test

The motor function of the animals was assessed before and at 1, 7, 14, 21, 28, 35, and 42 days after the onset of ischemia using a rotarod treadmill (Model MK-630B; Muromachi Kikai Co., Japan). The rotarod treadmill was set to the acceleration mode of 4 to 40 rpm for 5 min. Each animal (vehicle/direct injection group: *n* = 10, BMSC/direct injection group: *n* = 11, and BMSC/scaffold group: *n* = 8) was trained for 3 days before the study. The maximum time that the animal stayed on the rotarod was recorded for each performance, as described previously [11].

### 2.6. Histological Analysis

Five weeks after transplantation, the animals were deeply anesthetized with 4.0% isoflurane in N_2_O/O_2_ (70 : 30) and transcardially perfused with 20 mL of heparinized saline, followed by 50 mL of 4% paraformaldehyde. The brains were removed and 4 *μ*m thick coronal sections were prepared for subsequent analysis.

Fluorescence immunohistochemistry was performed to identify the donor-derived GFP-positive cells, as described previously [11]. A mouse monoclonal anti-GFP antibody (1 : 100 dilution; Santa Cruz Biotechnology Inc., Santa Cruz, CA, USA) and Zenon Alexa Fluor 488 fluorescent label (Molecular Probes Inc., Eugene, OR, USA) were used. The number of the GFP-positive cells that migrated toward the peri-infarct area was counted in five regions of interest (ROIs; 450 × 550 *μ*m) placed on the dorsal and ventral neocortex adjacent to the cerebral infarct (Figure 3(c)).

Double fluorescence immunohistochemistry was performed to evaluate the fate of the donor cells, as described previously [11]. Briefly, each section was treated with primary antibody against NeuN (mouse monoclonal, 1 : 100 dilution; Millipore Co., Billerica, MA, USA), and then it was labeled using a secondary antibody labeled with Alexa Fluor 594 (1 : 200 dilution; Molecular Probes). Subsequently, the sections were incubated with primary antibody against GFP tagged with Zenon Alexa Fluor 488 (Molecular Probes). Five ROIs were placed in the ipsilateral neocortex adjacent to cerebral infarct as described above. The percentages of the cells that were double positive for GFP and NeuN compared to the total GFP cell population were calculated (*n* = 5).

**Figure 4:**
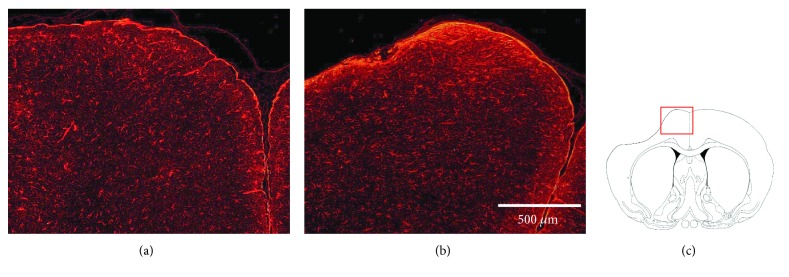
Immunohistochemistry for GFAP. Panels (a) and (b) show the fluorescence photomicrographs of GFAP 5 weeks posttransplantation in the BMSC/direct injection (a) and BMSC/scaffold (b) groups. A red rectangle in panel (c) indicates the location. Scale bars: 500 *μ*m.

Furthermore, fluorescence immunohistochemistry was performed to evaluate the influence of the RCP sponge on the brain surface using a primary antibody against glial fibrillary acidic protein (GFAP; mouse monoclonal, 1 : 500 dilution; BD Pharmingen, San Diego, CA, USA) and Alexa Fluor 594 (Molecular Probes). The activation of astrocytes around the brain surface was compared between the RCP sponge animals and direct injection animals. The ROIs (900 × 1100 *μ*m) were set in the ipsilateral dorsal neocortex (*n* = 6 in both groups; Figure 4(c)). Then, the area occupied by GFAP-positive cells was quantified with the image analysis system Image J 1.41 (National Institutes of Health, MD) in each ROI [10]. Briefly, the red fluorescence signal emitted from cells stained with the anti-GFAP antibody was binarized, and each area was presented as the percentage of the whole area of the ROI.

### 2.7. Statistical Analysis

All data were expressed as mean ± standard deviation (SD). Continuous data were compared using Student's *t*-test. Values of *P* < 0.05 were considered statistically significant.

## 3. Results

### 3.1. BMSCs in RCP Sponge In Vitro

The BMSCs could be cultured on RCP sponge and passaged twice, and living cells were found on RCP sponge 14 days after the second passage (Figure 1(c)). Scanning electron microscopy showed the RCP sponge had holes and the BMSCs adhered to the material, bridging the holes (×1500; Figure 1(d)). The cell viability was about 95% or higher on day 1, 7, and 14 postculture in RCP sponge (*n* = 3; Figure 1(e)).

### 3.2. Functional Recovery

**Figure 5:**
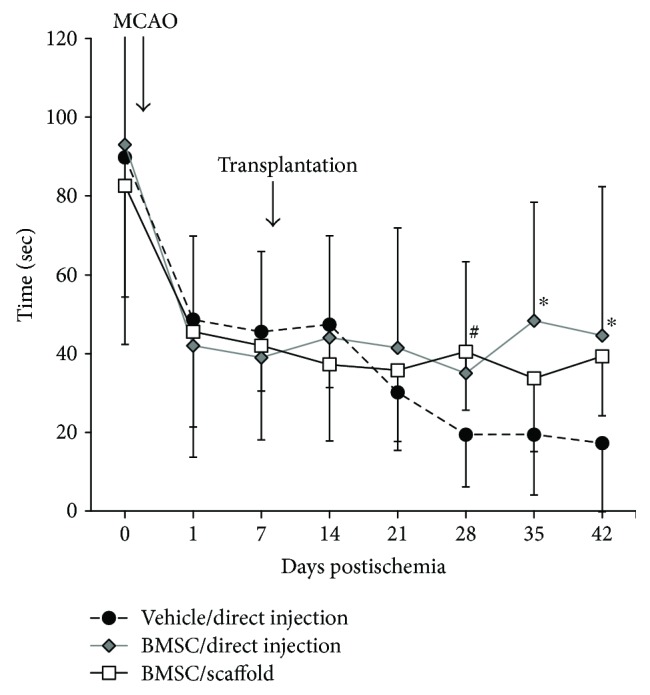
Rotarod test. The line graph shows the mean time on a rotarod treadmill for each group. Black circles: the vehicle/direct injection group; shaded diamonds: the BMSC/direct injection group; white squares: the BMSC/scaffold group. Error bars show SD; ^#^*P* < 0.05 in the BMSC/scaffold group versus the vehicle/direct injection group, ^∗^*P* < 0.05 in the BMSC/direct injection group versus the vehicle/direct injection group.

All animals survived the surgeries and were used for subsequent analyses. As shown in Figure 5, all animals exhibited severe neurological deficit one week after the onset of focal cerebral ischemia. There was no significant difference in motor function among the three groups 7 days postischemia. Subsequently, marked deterioration of motor function was found in the vehicle/direct injection group. In contrast, motor function significantly improved 28 and 35 days posttransplantation in the BMSC/direct injection group (*P* = 0.008 and 0.027, resp.). The animals in the BMSC/scaffold group showed significant improvement of motor function at 21 days posttransplantation (*P* = 0.042) and were similar to the BMSC/direct injection group at 28 and 35 days posttransplantation.

### 3.3. BMSCs in RCP Sponge In Vivo

All animals were sacrificed at 5 weeks posttransplantation and their brains were removed. In the BMSC/scaffold group, some RCP sponge remained on the brain (Figure 2(d)). The RCP sponge did not adhere to the brain surface and so was readily removed. Moreover, no abnormal findings were seen on the brain surface after the RCP sponge was removed.

### 3.4. Donor Cell Survival and Phenotypic Change

**Figure 6:**
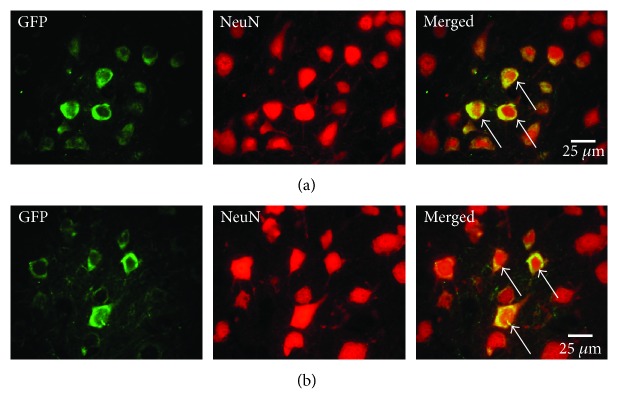
Immunohistochemistry for GFP and NeuN. Panels (a) and (b) show fluorescent photomicrographs adjacent to the cerebral infarct 5 weeks posttransplantation in the BMSC/direct injection (a) and BMSC/scaffold (b) groups. Green: GFP; red: NeuN. Arrows indicate coexpressing cells. Scale bars: 25 *μ*m.

GFP-expressing cells were extensively distributed adjacent to the cerebral infarct at 5 weeks posttransplantation in both the BMSC/direct injection and BMSC/scaffold groups. Some of the migrating GFP-positive cells in both groups expressed NeuN, a neuronal marker (Figure 6). Quantitative analysis indicated that the number of GFP-positive cells in the peri-infarct area was not different between the two groups (BMSC/direct injection group: 42.9 ± 4.0 cells/mm^2^ and BMSC/scaffold group: 39.0 ± 10.3 cells/mm^2^). The analysis of each ROI showed that the donor cells migrated to the peri-infarct area (ROI numbers 1, 2, 4, and 5) in the BMSC/scaffold group but not to the deeper brain parenchyma (ROI number 3), although there was no significant difference between the two groups (Figure 3(a)). The percentage of GFP and NeuN double-positive cells in the GFP-positive cell population was high (76.9 ± 14.1% in the BMSC/direct injection group and 91.8 ± 29.1% in the BMSC/scaffold group), but there was no significant difference between the ROIs or groups (Figure 3(b)).

### 3.5. Reactive Astrocytes in the Host Brain

**Figure 7:**
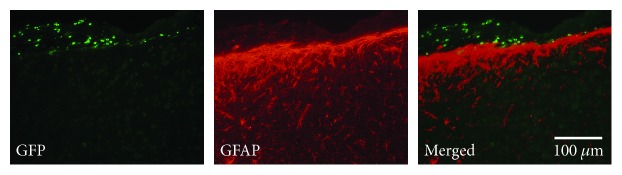
Immunohistochemistry for GFP and GFAP. Panels show fluorescent photomicrographs around the brain surface where the RCP sponge contacted 5 weeks posttransplantation in the BMSC/scaffold groups. Green: GFP; red: GFAP. Scale bars: 100 *μ*m.

Quantitative analysis of GFAP immunofluorescence showed no significant difference between the two groups (20.1 ± 3.8% in the BMSC/direct injection group and 22.7 ± 11.6% in the BMSC/scaffold group), although an increase in reactive astrocytes was found at the brain surface due to contact with the RCP sponge (Figure 4). In the BMSC/scaffold group, some BMSCs were found at the brain surface where the RCP sponge contacted, most of them did not coexpress GFAP (Figure 7).

## 4. Discussion

Our present study showed that BMSCs proliferated well on the RCP sponge. Compared with the vehicle group, the BMSC-RCP sponge construct promoted functional recovery posttransplantation onto the ipsilateral intact neocortex. Histological analysis revealed that the RCP sponge induced little inflammatory reaction in the host brain. Moreover, some BMSCs migrated to the peri-infarct area and differentiated into neuronal cells.

The RCP was designed using human collagen type I (*α* I chain) [12]. This type of collagen exists in large quantities in tissues such as dermis and bone. It is enriched with arginine-glycine-aspartate (RGD) sequences that enhance cell adhesion. RCP has high safety and biocompatibility because it is a nontoxic sequence of human type I collagen and does not contain animal-derived material. It is biodegradable and bioabsorbable, so it does not remain in the body. In order to demonstrate the biodegradability in vivo for RCP, the hydrogel made of RCP was implanted under the dorsal skin of mice. The results indicated that the dry weight was 55% on day 5 and 9% on day 14 to the time of implantation. Thus, RCP is going to decompose gradually in vivo [12]. Moreover, RCP has an extremely uniform molecular weight distribution of approximately 51 kD and is flexible, so it can be formulated into various forms, such as sponges, porous particles, and granules.

Tissue engineering involves implantation of a scaffold made with biomaterials and seeded with transplanted cells. Unlike surgical materials made from polymers, ceramics, or titanium, the biomaterials used in regenerative medicine must be biodegradable, porous, and cytophilic. Such biomaterials have been used for cell therapy in the bone, cartilage, blood vessels, heart, and skin. However, these organs are less complex than the CNS [14]. Although cell therapy using scaffolds for the CNS has many limitations, the RCP sponge seems to be suitable for use due to its properties. The supplier of the material showed that cell proliferation was equivalent to BMSCs cultured using collagen 1-coated flasks (data not shown), and if the pore size of the material can be optimized, the cell proliferative capacity may increase. In the present study, it was found that reactive astrogliosis increased slightly near the brain surface in contact with the RCP sponge. As the sponge is a human collagen-based material, it may cause astrogliosis in rat brains but not human brains. Moreover, the RCP sponge was present on the brain surface at the time of animal sacrificed, and astrogliosis may become weaker after the material degrades over time in vivo. In the present study, although we had 5-week observation after the transplantation, it seems that a longer duration study would be beneficial for verification of astrogliosis and degradation of the material in BMSC/scaffold group.

In the present study, the animals in the BMSC/scaffold group showed significant improvement of motor function 21 days posttransplantation but not at 28 and 35 days, compared with the vehicle group. In contrast, motor function improved 28 to 35 days posttransplantation in the BMSC/direct injection group. Thus, the BMSC-RCP sponge construct promoted functional recovery in the early phase after transplantation, but the therapeutic potential appeared to be weaker in the later phase. In the early phase, there is a “nursing effect,” which is a neuroprotective effect caused by paracrine secretion of cytokines or neurotrophic factors from donor cells and that plays an important role in the therapeutic potential of the cells [15]. The BMSC-RCP sponge construct may provide a greater number of BMSCs to the peri-infarct area than direct cell injection, promoting an earlier therapeutic effect in the BMSC/scaffold group. However, most BMSCs at the peri-infarct area would have died in early phase, except for cells that integrated into the host brain, such as cells expressing neural markers [15]. Further, it is difficult for the donor cells in BMSC/scaffold group to reach the deeper brain parenchyma, although they can readily migrate to the peri-infarct area.

Neurogenesis can occur in the subventricular zone (SVZ) of the adult rodent brain [16] and the directly transplanted BMSCs may migrate to the SVZ and enhance intrinsic neurogenesis in the host brain, as we showed in a previous study. Interestingly, some donor cells, which did not express any neural markers, survived in the ependymal layer in contact with neural stem/neuronal precursor cells. We proposed that the donor cells that had not differentiated into neurons integrated into the ependymal layer and stimulated intrinsic neurogenesis through a paracrine mechanism [17]. In the present study, fewer donor cells were found around the SVZ (ROI 3 in Figure 3) in the BMSC/scaffold group than in the BMSC/direct injection group. Moreover, fewer nonneuronal donor cells were found around the SVZ in the BMSC/scaffold group. Thus, the therapeutic potential may be weaker in later phases because the potential to stimulate intrinsic neurogenesis around the SVZ was lower in the BMSC/scaffold group. But we did not find whether the therapeutic potential remained weaker or not in the BMSC/scaffold group. A follow-up study would need to be performed to answer this issue.

The direct cell transplant technique is an invasive method, though it can deliver the cells to the deep brain region. The BMSC-RCP sponge construct, on the other hand, has great potential to deliver many cells from the surface of brain with less invasion, but it would be hard for the cells to reach the deep region, especially in human brain. Although this is a limitation of not only the BMSC-RCP sponge construct but also the scaffold set on the surface of brain in general, it may be solved with the concomitant use of additional less invasive methods, such as intrathecal, intra-arterial, or intravenous administration of cell suspensions. These combined cell delivery methods might be effective to deliver many cells from both inside and outside of the brain with less-invasion. Thus, the combined method may be useful as an option because it could make up for the shortcomings of the BMSC-RCP sponge construct.
